# Integration of AI in Diagnostic Methods for Dementia -A Scoping Review

**DOI:** 10.1192/bjo.2026.11127

**Published:** 2026-06-30

**Authors:** Natasha Singh, Alexandra Minseo Kim

**Affiliations:** 1 Darent Valley Hospital, Dartford, United Kingdom; 2 University of Lancashire, Preston, United Kingdom

## Abstract

**Aims:**

To discuss the potential role of artificial intelligence (AI) in different diagnostic interventions in dementia.

**Methods:**

A literature search was conducted in PubMed on 6 February 2026 using the terms “*artificial intelligence*” AND “*dementia*”. The search identified 5257 records, which were limited to publications from 2021–2026, yielding 3598 records. Filters for free full-text availability and study type (clinical trials, randomized controlled trials, systematic reviews, meta-analyses, and reviews) were applied, resulting in 303 articles.

Inclusion criteria were: (1) studies focused on the application of artificial intelligence in dementia; (2) human studies; (3) peer-reviewed articles; (4) publication within the last five years; and (5) availability of full text in English. Exclusion criteria included: (1) non-dementia populations; (2) non-AI-based methodologies; (3) conference abstracts, editorials, letters, and commentaries; and (4) animal or simulation-only studies.

Titles of the 303 eligible articles were screened for relevance, resulting in 50 studies included for further analysis.

**Results:**

Dementia is a clinical outcome of complex neurological disorders with a multitude of aetiologies and risk factors. Conventional methods in early diagnosis and management of dementia pose several risks and costs. Integrating AI with a sophisticated learning model can provide a comprehensive assessment in the diagnosis of dementia. Early diagnostic efforts can be achieved through a combination of AI-based neuroimaging such as MRI, PET, and CT scans, genetic and metabolic biomarkers supported by a robust pattern recognition algorithm. Incorporation of AI in dementia diagnosis can enable early recognition, leading to a 40% risk modification.

However, a number of methodological constraints should be noted when using AI for diagnosis like lack of algorithm development and standard definitions. Other challenges include difficulties in integrating healthcare systems, as well as data privacy and ethical concerns.

**Conclusion:**

Artificial intelligence has significant potential to enhance the diagnosis and prognosis of dementia by improving early detection and reducing costs. Despite methodological, ethical, and integration challenges, effective collaboration between AI and medical professionals and the development of standardized approaches are essential for successful implementation in clinical practice.

